# Distribution and inflammatory cell response to intracranial delivery of radioluminescent Y2(SiO_4_)O:Ce particles

**DOI:** 10.1371/journal.pone.0276819

**Published:** 2023-01-12

**Authors:** Máté Fischer, Amber Zimmerman, Eric Zhang, Joseph Kolis, Ashley Dickey, Mary K. Burdette, Praveen Chander, Stephen H. Foulger, Jonathan L. Brigman, Jason P. Weick

**Affiliations:** 1 Department of Neurosciences, University of New Mexico HSC, Albuquerque, New Mexico, United States of America; 2 Department of Materials Science and Engineering, Clemson University, Clemson, South Carolina, United States of America; 3 Center for Optical Materials Science and Engineering Technologies, Clemson University, Clemson, South Carolina, United States of America; 4 Department of Bioengineering, Clemson University, Clemson, South Carolina, United States of America; 5 Center for Brain Recovery and Repair, University of New Mexico HSC, Albuquerque, New Mexico, United States of America; Indian Institute of Technology Delhi, INDIA

## Abstract

Due to increasing advances in their manufacture and functionalization, nanoparticle-based systems have become a popular tool for *in vivo* drug delivery and biodetection. Recently, scintillating nanoparticles such as yttrium orthosilicate doped with cerium (Y2(SiO_4_)O:Ce) have come under study for their potential utility in optogenetic applications, as they emit photons upon low levels of stimulation from remote x-ray sources. The utility of such nanoparticles *in vivo* is hampered by rapid clearance from circulation by the mononuclear phagocytic system, which heavily restricts nanoparticle accumulation at target tissues. Local transcranial injection of nanoparticles may deliver scintillating nanoparticles to highly specific brain regions by circumventing the blood-brain barrier and avoiding phagocytic clearance. Few studies to date have examined the distribution and response to nanoparticles following localized delivery to cerebral cortex, a crucial step in understanding the therapeutic potential of nanoparticle-based biodetection in the brain. Following the synthesis and surface modification of these nanoparticles, two doses (1 and 3 mg/ml) were introduced into mouse secondary motor cortex (M2). This region was chosen as the site for RLP delivery, as it represents a common target for optogenetic manipulations of mouse behavior, and RLPs could eventually serve as an injectable x-ray inducible light delivery system. The spread of particles through the target tissue was assessed 24 hours, 72 hours, and 9 days post-injection. Y2(SiO_4_)O:Ce nanoparticles were found to be detectable in the brain for up to 9 days, initially diffusing through the tissue until 72 hours before achieving partial clearance by the final endpoint. Small transient increases in the presence of IBA-1^+^ microglia and GFAP^+^ astrocytic cell populations were detected near nanoparticle injection sites of both doses tested 24 hours after surgery. Taken together, these data provide evidence that Y2(SiO_4_)O:Ce nanoparticles coated with BSA can be injected directly into mouse cortex *in vivo*, where they persist for days and are broadly tolerated, such that they may be potentially utilized for remote x-ray activated stimulation and photon emission for optogenetic experiments in the near future.

## Introduction

Numerous nanoparticle-based systems have been proposed in recent years to address a broad range of therapeutic and diagnostic aims [1–3]; given the substantial promise of this emerging technology, concerns surrounding the delivery, diffusion, tolerance and clearance of nanoparticle-based systems leave many unresolved questions [4, 5]. Nanoparticles generated from a variety of core materials display high variability in their biocompatibility and utility [2]. Silicon-based nanoparticles have been extensively probed for potential biomedical applications due to their low inherent toxicity, especially when administered chronically [6]. Additionally, wide band gap materials doped with rare earth ions represent an active area of research regarding their applications in medical imaging, such as their use in the development of PET detectors [7]. More recently, a novel class of scintillating nanoparticles, rare earth orthosilicates such as yttrium orthosilicate doped with cerium Y2(SiO_4_)O:Ce, have been under investigation for imaging and light delivery applications [8, 9].

Scintillating nanoparticles provide a platform for optically-based biomedical applications because of their ability to emit photons upon low level x-ray stimulation, which can be detected through multiple imaging modalities. Scintillating materials such as Y2(SiO_4_)O:Ce could also allow investigators to deliver specific emission spectra of light to targeted tissues allowing for optogenetic manipulations in biological systems [10, 11]. Furthermore if biologically tolerated, the blue light emission spectrum of Y2(SiO_4_)O:Ce, may provide a novel injectable light delivery system for optogenetic uses in the brain, where commonly used wavelengths of light are severely limited by light scatter [12, 13].

Currently, *in vivo* optogenetic methods require the use of an implantable optical fiber into target tissues expressing a genetically encoded bacterial opsin such as Channelrhodopsin-2 (ChR2), a 7 transmembrane domain channel protein capable of excitation upon stimulation with specific wavelengths [14]. Scintillating nanoparticles could potentially be designed to emit tunable spectra of visible light in order to replace currently available approaches based on surgical implantation of optical fibers. Cell type-specific expression of optically-activated constructs paired with localized light stimulation from scintillating nanoparticles would provide a far less invasive and more targeted platform for optogenetic experiments.

In a previous meta-analysis, Wilhelm *et al*. demonstrated that only approximately 0.7% of systemically injected nanoparticles reach target tumors by passive diffusion, which limits the efficacy of these particles to barely appreciable levels [15]. In addition to the shortcomings inherent to passive diffusion, the mononuclear phagocytic system also represents a significant barrier to the focal delivery of particles to target tissues following systemic administration [16]. In this report, we stereotaxically injected cerium-doped nanoparticles coated with BSA and fluorescein into mouse secondary motor cortex, in order to track nanoparticle dispersion and persistence in the target tissue over time, as well as to quantify aspects of the immune cell recruitment following the injection of nanoparticles. We found that scintillating nanoparticles could reproducibly be delivered to M2, where they were readily detectable up to 9 days post-injection.

## Materials and methods

### Nanoparticle production

#### Reagents and solvents

All reagents were purchased from Alfa Aesar, Sigma Aldrich, VWR, and J.T. Baker and were used without purification. All solvents used for reactions were dried according to standard methods. Deionized water was obtained from a Nanopure system and exhibited a resistivity of 18.2 MOhm-cm. Column chromatography was performed using silica gel (spherical neutral, particle size 63–210 μm).

#### Chemical characterization

H NMR spectra were recorded on a JEOL ECX300 spectrometer. Chemical shifts for protons are reported in parts per million downfield from tetramethylsilane and referenced to residual protium in the NMR solvent (CDCl_3_: δ 7.26 ppm).

#### Synthesis of yttrium orthosilicate nanoparticles

Sub-100 nm silica cores were synthesized by the sol-gel process with 27 mmol (6.0 ml) of tetra ethyorthosilicate, 167 mmol (3 ml) of water, and 50 mmol (2 ml) of ammonia hydroxide (28% v/v) in 150 ml of ethanol at room temperature under vigorous stirring condition for 20 hours. The particles were then washed with ethanol (2x) and sterile water (1x). Silica (120 mg) was dispersed in sterile water (100 ml) with 1.98 mmol (760 mg) of yttrium nitrate hexahydrate and 15 μmol (6.5 mg) of cerium nitrate hexahydrate. Ammonia hydroxide (1 ml) was added dropwise into the solution and vigorously stirred for 16 hours at room temperature. The particles were washed with water (3x) and dried at 125°C. The particles were then annealed at 1000°C for 1 hour.

#### Synthesis of 2,5-dioxopyrrolidin-1-yl hept-6-ynoate (alkNHS)

**Fig 1:**
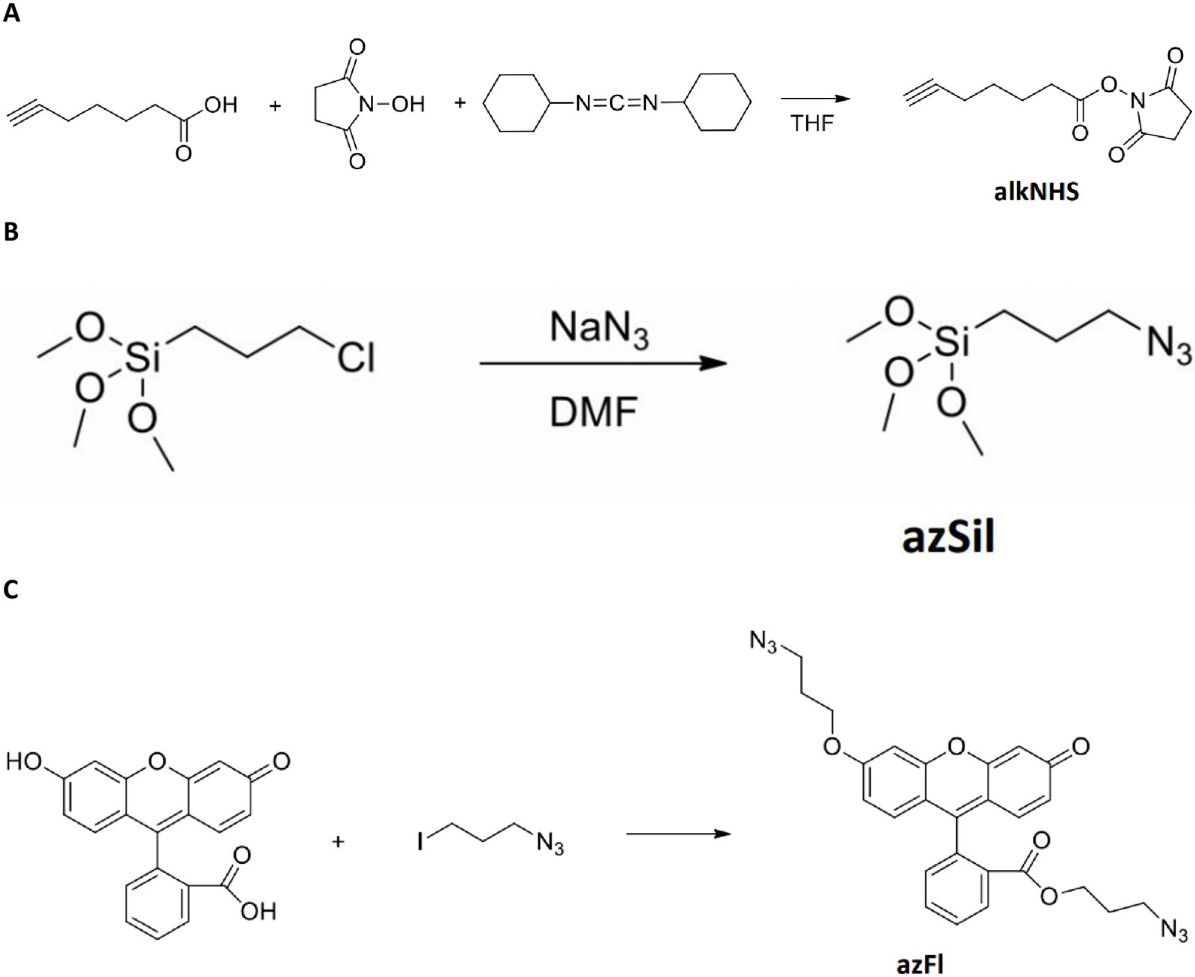
Synthesis of chemical intermediates for enhanced biocompatibility and tracking of nanoparticles in vivo. Synthetic schemes to yield (a) alkNHS, (b) azSil and (c) azFl.

2,5-dioxopyrrolidin-1-yl hept-6-ynoate (alkNHS) was synthesized according to the chemical formula illustrated in Fig 1A. First, hept-6-ynoic acid (1 g, 7.93 mmol) and N-hydroxysuccinimide (1 g, 8.72 mmol) were dissolved in Tetrahydrofuran (THF, 25 ml). The solution was stirred and cooled with ice. Once cool, dicyclohexylcarbodiimide (1.8 g, 8.72 mmol) was added and the obtained mixture was stirred with cooling for 15 minutes. The mixture was then stirred at room temperature for 24 hours and then filtered through a 0.45μm syringe filter (Sigma-Aldrich). The insoluble solid was washed with dichloromethane and the filtrate was evaporated under reduced pressure. The residue was dissolved in dichloromethane and the obtained solution was washed with water. The organic layer of this solution was separated and dried with Na_2_SO_4_, filtered as above, and evaporated under reduced pressure. The residue was dissolved in dichloromethane (10 ml) and cooled. Insoluble impurities were separated by filtration and the filtrate was evaporated to give a product with 90% purity by NMR. Yield 1.55 g, clear solid, mp = 55–56°C. ^1^H NMR (CDCl_3_) δ 1.65 (m, 2H, J = 7.0 Hz), 1.87 (m, 2H, J = 7.6 Hz), 1.97 (t, 1H, J = 2.4 Hz), 2.25 (m, 2H, J = 7.0 Hz, J = 2.4 Hz), 2.65 (t, 2H, J = 7.6 Hz), 2.84 (s, 4H).

#### Preparation of alkyne-labeled bovine serum albumin (alkBSA)

A 0.001 M solution of BSA (500 mg, 7.52 μmol) in 1x PBS along with a 0.021 M solution of alkNHS (2.34 mg, 10.5 μmol) in THF were added to a test tube. The resulting solution was shaken via wrist-action shaker for 24 hours in the dark. After 24 hours the solution was transferred to a float-a-lyzer dialysis bag (8000–10000 MWCO) and dialyzed in D.I. H_2_O for 3 days with water changed frequently. The resulting clean alkBSA was stored at 4°C in the dark at a concentration of 0.658 mM in 1x PBS.

#### Synthesis of (3-azidopropyl)trimethoxysilane (azSil)

(3-Azidopropyl)trimethoxysilane was synthesized by a previously reported procedure [17], which is illustrated in Fig 1B. (3- Chloropropyl)trimethoxysilane (1 g, 5.03 mmol) and sodium azide (0.684 g, 10.1 mmol) were mixed with dry dimethylformamide (3 ml) under nitrogen. The obtained mixture was stirred at 90°C for 1 hour. After cooling, the mixture was filtered as described above. The filtrate solution contained 25% of product in DMF and was used in the next step.

#### Azide-modified Y2(SiO4)O:Ce (sub-100nm) particles (azY2(SiO4)O:Ce)

Sub-100 nm Y2(SiO_4_)O:Ce particles were modified through organometallic chemistry using azSil [18, 19]. Y2(SiO_4_)O:Ce particles (200 mg) were mixed with DMF (6 ml) in a 50 ml round bottom equipped with a stir bar. The obtained suspension was sonicated until well dispersed (~ 1 minute). A solution of azSil (20 μl, 25% in DMF) was added along with D.I. H_2_O (0.4 ml) and the mixture was stirred for 1 minute. Finally, an aqueous solution of ammonium hydroxide (40 μl, 29% solution) was added to the mixture and stirred for 2 hours. The particles were washed with ethanol via centrifugation in a Sorvall Legend X1R (5 min. x 4284g) and dried under vacuum at room temperature (3x). The resulting azY2(SiO_4_)O:Ce particulates were stored at room temperature.

#### Synthesis of 3-azidopropyl 2-(6-(3-azidopropoxy)-3-oxo-3H-xanthen-9-yl) benzoate (azFl)

Chemical synthesis of (3-azidopropyl 2-(6-(3-azidopropoxy)-3-oxo-3H-xanthen-9-yl) benzoate (azFl) is illustrated by the formula in Fig 1C. Fluorescein (FITC, 0.4 g, 1.2 mmol) and 1-azido-3-iodopropane (0.56 g, 2.65 mmol) were dissolved in dry DMF (5ml). Potassium carbonate (0.5g, 3.6 mmol) was added to the solution under nitrogen atmosphere. The resulting mixture was heated to 80°C and stirred for 4 hours. After cooling, the mixture was extracted with dichloromethane and washed with water. The organic layer was separated by evaporation under vacuum. The resulting crude residue was mixed with water and the insoluble product was separated by decantation, dried under vacuum, and purified by column chromatography with silica. Solvent methanol:dichloromethane (1:20), Rf = 0.2. Yield 0.55 g (92%), orange crystals, m.p. = 86–87°C. 1H NMR (CDCl_3_) δ 1.62 (m, 2H, J = 6.2 Hz), 2.10 (m, 2H, J = 6.2 Hz), 306 (t, 2H, J = 6.2 Hz), 3.54 (t, 2H, J = 6.2 Hz), 4.08 (t, 2H, J = 6.2 Hz), 4.16 (t, 2H, J = 6.2 Hz), 6.45 (d, 1H, J = 2.4 Hz), 6.54 (d.d, 1H, J = 2.4 Hz, J = 9.6 Hz), 6.75 (d.d, 1H, J = 2.4 Hz, J = 8.9 Hz), 6.85(d, 1H, J = 9.6 Hz), 6.89 (d, 1H, J = 8.9 Hz), 6.97 (d, 1H, J = 2.4 Hz), 7.31 (d.d, 1H, J = 1.4 Hz, J = 7.6 Hz), 7.65–7.78 (m, 2H, J = 1.4 Hz, J = 7.6 Hz), 8.25 (d.d, 1H, J = 1.4 Hz, J = 7.6 Hz).

#### Synthesis of BSA-coated Y2(SiO4)O:Ce particles tagged with azFl (Y2(SiO4)O/BSA/Fl)

azFl-tagged, alkBSA-coated, sub-100 nm azY2(SiO_4_)O:Ce particles were synthesized using a multistep copper(I) catalyzed azide–alkyne cycloaddition (CuAAC) reaction. AlkBSA (50 mg in 1.14 ml 1x PBS) and a 0.0016 M aqueous solution of copper(II) sulfate (0.375 mg, 1.5 μmol) were added to a test tube equipped with a stir bar. A 0.004 M aqueous solution of sodium ascorbate (0.745 mg, 3.76 μmol) was added to the test tube followed by a 0.0038 M solution of azFl (2.81 mg, 5.64 μmol) in THF. The constituents were added such that the volume of THF in the test tube never exceeded 40% volume. The reaction was allowed to react with stirring at 28°C, under nitrogen, and in the dark for 1 hour. After 1 hour, a suspension of azY2(SiO_4_)O:Ce (100 mg in 1 ml 1x PBS) was added to the test tube. The reaction was allowed to continue reacting with stirring at 28°C, under nitrogen, and in the dark for 23 hours. The resulting suspension was cleaned via centrifugation with a solution of 1x PBS and THF at a ratio of 60:40 (v/v) until the supernatant showed no free dye. The resulting solution of Y2(SiO_4_)O/BSA/Fl (radioluminescent particles, RLPs) was stored in 5 ml 1x PBS at 4°C.

#### Characterization of core-shell yttrium orthosilicate nanoparticles

Sub-100 nm particulates were dispersed in water on double-sided carbon tape and mounted onto a scanning electron microscope stub. The particles were plasma coated with platinum. A Hitachi 4800 scanning electron microscope was used to image the particles at 20 kV and 10 mA. Photoluminescence emission spectra were taken by a Jobin Yvon Fluorolog 3–222 spectrometer with a excitation source of 360 nm (450W Xe lamp). X-ray luminescence was measured with a Horiba JY synapse CCD camera, Horiba JY microHR monochromator, and a tungsten target Amptek mini x-ray source at 40 kV and 99 μA.

### Biodistribution, inflammatory, and toxicity analyses

#### Experimental subjects

Female C57BL/6J mice (The Jackson Laboratory, Bar Harbor, ME) were housed in a temperature- and humidity-controlled vivarium under a reverse 12 hour light/dark cycle (lights off 0800 h). All experimental procedures were performed in accordance with the National Institutes of Health Guide for Care and Use of Laboratory Animals and were approved by the University of New Mexico Health Sciences Center Institutional Animal Care and Use Committee.

#### Microinjection of nanoparticles

At approximately 6 months of age, mice were anesthetized with isoflurane and fixed in a stereotaxic apparatus (1900 Stereotaxic Alignment System, David Kopf Instruments, Tujunga, CA) as previously described [20]. A 33-gauge infusion cannula (Plastics One, Roanoke, VA) attached with polyurethane tubing to a Hamilton syringe (Hamilton, Reno NV) was directed at secondary motor cortex (M2. AP: 1.345, ML: +/- 0.6, DV: − 1.0). Y2(SiO_4_)O-BSA-fluorescein nanoparticles (0.5 μl) or saline vehicle was infused over 3 min using a pump (GenieTouch, Kent Scientific, Torrington, CT), with the cannula left in place for an additional 1 minute to allow diffusion. Twelve mice were assigned to four groups (n = 3 per group): 3 mg/ml (one hemisphere) + saline (other), 1 mg/ml (one hemisphere) + saline (other) and sacrificed at 24 hours. For mice sacrificed at 72 hours or 9 days, they were provided bilateral injections of nanoparticles (3 mg/ml in one hemisphere + 1 mg/ml other hemisphere).

#### Immunohistochemistry

Six unilaterally-injected animals were sacrificed 24hr after injection, while 3 animals that received bilateral injections were sacrificed 72hr later, and the final 3 animals were sacrificed 216hr (9 days) following injection. All mice were sacrificed by cervical dislocation, brains were extracted and placed in 4% paraformaldehyde solution for 24 hours. 50 μm coronal sections were cut with a vibratome (Classic 1000 model, Vibratome, Bannockburn, IL) adjacent to the site of injection. Slices were blocked and permeabilized for 1 hour on a Belly Dancer shaking platform (IBI Scientific) in a 500 μl solution of 10% donkey serum, and 0.5% Triton X-100, brought to volume with 1x PBS. After one hour incubation at room temperature the blocking solution was removed and replaced with 500 μl of primary antibody solution containing monoclonal mouse anti-GFAP (dilution 1:500; Neuromab, USA) and polyclonal rabbit anti-IBA-1 (dilution 1:250; Wako, Japan) diluted in 5% donkey serum and 0.25% Triton X-100 brought to volume in PBS. Sections were covered and incubated in a 4°C walk-in cold room overnight.

The following day, the primary antibody solution was removed, and slices were washed (3x) with 500μL PBS for 5 minutes each. Following the three washes, 500μL of secondary antibody solution containing donkey anti-mouse AlexaFluor 647 (dilution 1:500; Abcam, USA) and donkey anti-rabbit AlexaFluor 550 (dilution 1:500; Abcam, USA) diluted in 5% donkey serum and 0.25% Triton X-100 brought to volume in PBS was added to the sections at room temperature for 1 hour. After this incubation the secondary antibody solution was removed, and slices were washed 3 times with 500 μl 1x PBS for 5 minutes each. DAPI counterstaining (1:10000 concentration in PBS) was then performed and slices were placed on a rocker for 10 minutes shielded from light. Finally, the slices were washed with 500 μl PBS (3x) for 5 minutes each and mounted to glass microscope slides. Slices were covered with 100 μl of Fluoromount-G (Thermo Fischer Scientific, USA) and then sealed with glass coverslips, making sure to remove any air bubbles. Slides were finally stored in a 4°C refrigerator for subsequent imaging.

#### Confocal microscopy

Following immunostaining and mounting, nanoparticle-associated fluorescein fluorescence-labeled immune cell populations were imaged using a Leica TCS SP8 Confocal Microscope (Leica Microsystems). DAPI, nanoparticle-conjugated fluorescein and secondary antibody-conjugated fluorophores were excited using a 405nm laser diode and a tunable pulse white light laser, respectively, with a 10x air objective and a 63x oil immersion objective and captured using hybrid spectral detectors (HyD). Z-stacks were then collected with a Z-range of 70 μm. Z stacks of adjacent slices were ordered and merged to estimate the extent of the nanoparticle spread and compressed then into a two dimensional image using the maximal intensity projection algorithm in the Leica LASX software package. Maximum projection maps were exported as uncompressed TIFF files and transferred to ImageJ image analysis software.

#### Analysis and statistics

Optical sections of 25 μm thickness were collected for the brains that were sectioned and stained for quantification of nanoparticle-fluorescein, IBA-1 and GFAP signals, respectively. After Z stacks were compressed, ImageJ software was used to draw regions of interest (ROIs) surrounding the entire extent of the M2 region with the help of the Allen Brain Atlas for the given appropriate A/P range. Light signal from photon scatter around the edges of tissue, tears in the tissue as wells as vasculature were excluded from analysis. Mean fluorescence intensities were calculated over the ROIs, and a background measurement taken from cortical regions outside M2 was subtracted from these values to normalize for the autofluorescent and nonspecific signal present in the sample. A ratio was then calculated for signal intensity in the nanoparticles vs. vehicle treated hemispheres, mean differences were compared using student’s *t* test, and were considered significant a priori if *p* < 0.05. ImageJ was scaled such that the pixel to mm ratio was defined for each image and using this scale, the area of the extent of nanoparticle spread was calculated by manually tracing the outlines of the fluorescein signal adjacent to the site of injection.

#### Hippocampal neuron cultures and *in vitro* toxicity assay

Primary hippocampal neurons were cultured according to previously published methods [21]. Briefly, brains were removed from P0-P1 mouse pups and hippocampi were microdissected, dissociated in Trypsin (0.25%; Thermo Fisher Scientific), and plated at a density of 4–5 x 10^5^ cells/12mm glass coverslips. Primary neurons were maintained in 500μl of phenol-red-free neurobasal media (Thermo Fisher Scientific) with supplements described previously [21]. Replicates consisting of four individual 12mm coverslips per group (24hr, 72hr, and 216hr) received Y2(SiO_4_)O-BSA-fluorescein nanoparticles on the days indicated (see Results section). To allow for greater dispersion of particles across the entire coverslip and account for lower density of neurons, particles were diluted 1:100 (e.g. 1mg/ml to 0.01mg/ml and 3mg/ml to 0.03mg/ml) in feeding media, and then 100x volume that was infused in vivo (e.g. 50μl instead of 0.5μl) of particle-containing media was added in a spiral pattern to disperse particles evenly across coverslips.

We used the alamarBlue assay (Thermo Fisher Scientific) to assess metabolic activity of primary neurons according to the manufacturer’s specifications. Briefly, we added 50ul of 10x alamarBlue reagent directly to the media on all coverslips treated with RLPs, four control coverslips that received no particles, and four additional wells that contained media only and no cells. All wells were incubated for two hours, after which culture plates underwent absorbance measurements on a Tecan Infinite 200 plate reader at 570nm (experimental) and 600nm (control) wavelengths. Absorbance values from wells lacking cells were subtracted from experimental well across replicates, and 600nm reference values were subtracted from 570nm values. Resultant absorbance values were then used to calculate mean and standard error of the mean, and students *t* tests were used to compare means across groups.

## Results

### Generation and characterization of radioluminescent particles

**Fig 2:**
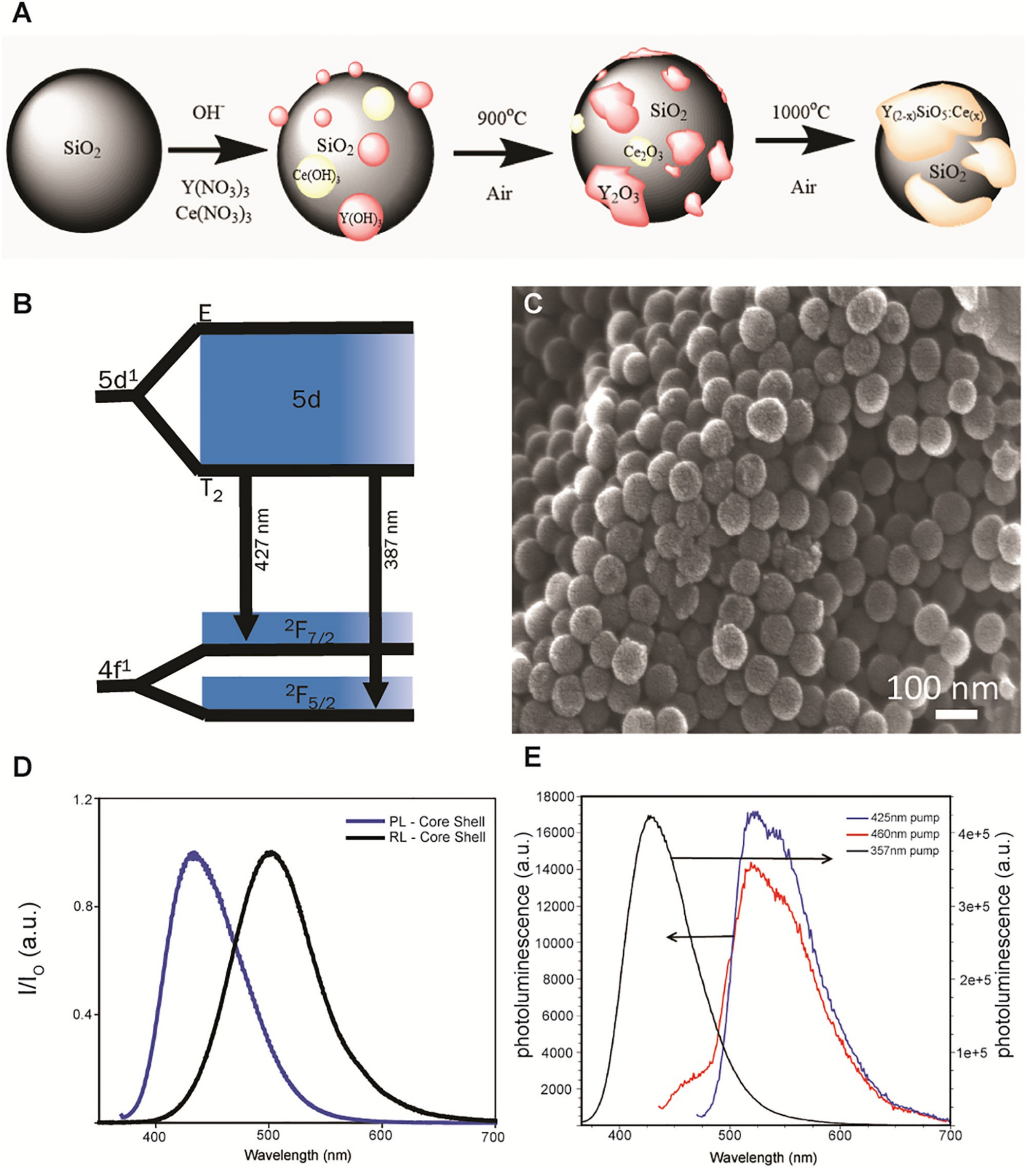
Generation and characterization of radioluminescent particles. A) Schematic of the development of Y2(SiO_4_)O:Ce using a core-shell method. B) Relaxation of cerium from it higher energy 5d state to its respective ground state of 2F7/2 and 2F5/2 in the Ce 1 site. C) Scanning electron micrograph of Y2(SiO_4_)O:Ce nanoparticles synthesized by the Stöber process. D) Photoluminescence and radioluminescence of nano core-shell particulates irradiated by 360 nm and a broadband 40 KeV tungsten x-ray source, respectively. E) Photoluminescence spectra of sub-100 nm Y2(SiO_4_)O/BSA/fluorescein nanoparticles in 1x PBS. A 100 μL sample of a 31.4 mg/ml dispersion was placed in 2.90 ml 1x PBS. The normal fluorescein peak at ca. 525 nm is present when the excitation wavelength is 425 nm (red) or 460 nm (blue). Also presented is the system excited at 357 nm (black) to show that the Y2(SiO_4_)O component is present.

A silica size template was synthesized using the Stöber process, in which different rare-earth hydroxides were made to precipitate onto the surface of particles to produce a core-shell morphology. Lightly annealing the core-shell particulates at 1000°C for 1 hour allowed for the core and the shell compounds to recrystallize to Y_2_SiO_5_ doped with cerium (Y2(SiO_4_)O:Ce) as depicted in Fig 2A. Cerium has an energy diagram as depicted in Fig 2B, where the ground state is split into 2F5/2 and 2F7/2 due to spin orbit coupling and its lowest excited energy state is in the 5d manifold. Significant yields of regular, spherical, sub-100nm Y2(SiO_4_)O:Ce particles can be obtained using this method (Fig 2C). Upon excitation at 360 nm light, a narrow emission band at 420 nm was observed due to the relaxation from the 5d state (Fig 2D), which is useful for excitation of fluorophores as well as microbial opsins [39]. Additionally, a bathochromic shift to 510 nm was observed under x-ray stimulation.

Addition of bovine serum albumin (BSA) to nanoparticles allows for greater biocompatibility as well as increased quantum efficiency of fluorescent substrates [22]. Azide functionalizing inorganic nanoparticles through organometallic chemistry such that they may participate in azide/alkyne click transformations with modified organic, biologically compatible moieties holds great promise for the use in biological applications [23, 24]. In the current report, a copper(I) catalyzed azide-alkyne cycloaddition (CuAAC) click reaction is used to introduce 1,2,3 triazole linkers to attach alkyne modified BSA to a sub-100 nm particulate scintillator, Y2(SiO_4_)O:Ce. Alkyne modified BSA can participate in a click transformation with an azide modified organic fluorophore prior to nanoparticle attachment to yield a BSA coated inorganic nanoparticles that can be identified and monitored in biological systems. We utilized a fluorescein moiety (FITC) to label the BSA coating of the inorganic nanoparticles to act as a visual reporter of the particles *in vivo* or *in vitro*.

A multistep CuAAC click reaction was employed to produce fluorescein labeled, BSA-coated Y2(SiO_4_)O:Ce. The BSA protein was modified with alkyne functionality using standard N- Hydroxysuccinimide (NHS) chemistry [25, 26]. In this system, half of the alkyne modified end groups of the functionalized BSA (alkBSA) were exploited for fluorophore labeling, while the other half were designated to coat the particulate scintillator (Y2(SiO4)O:Ce). The initial step of the click reaction was performed in a 40% THF, 60% 1x PBS/H2O solution for 1 hour and allowed for an azide modified fluorescein derivative to attached to approximately half of the available alkyne terminated sites of the alkBSA. After 1 hour, an aqueous dispersion of azide modified Y2(SiO_4_)O:Ce (azY2(SiO_4_)O:Ce) particles was added to the click reaction and allowed to react for 23 hours resulting in fluorescein labeled, BSA coated, Y2(SiO_4_)O:Ce particulate system (referred to here as radioluminescent particles (RLPs). To confirm the system’s functionality, the particulates were dispersed in 1x PBS and excited with wavelengths known to excite both the fluorophore and radioluminescent inorganic species shown in Fig 2E. When excited with 357 nm light, the particulate system demonstrates the normal Y2(SiO_4_)O:Ce photoluminescent emission near 420 nm exhibiting a blue color. However, when stimulated with 425 nm and 465 nm light, the particulate system demonstrated the normal fluorescein photoluminescent emission peak near 525 nm.

### Verification of injection site and radioluminescent particle detection

**Fig 3:**
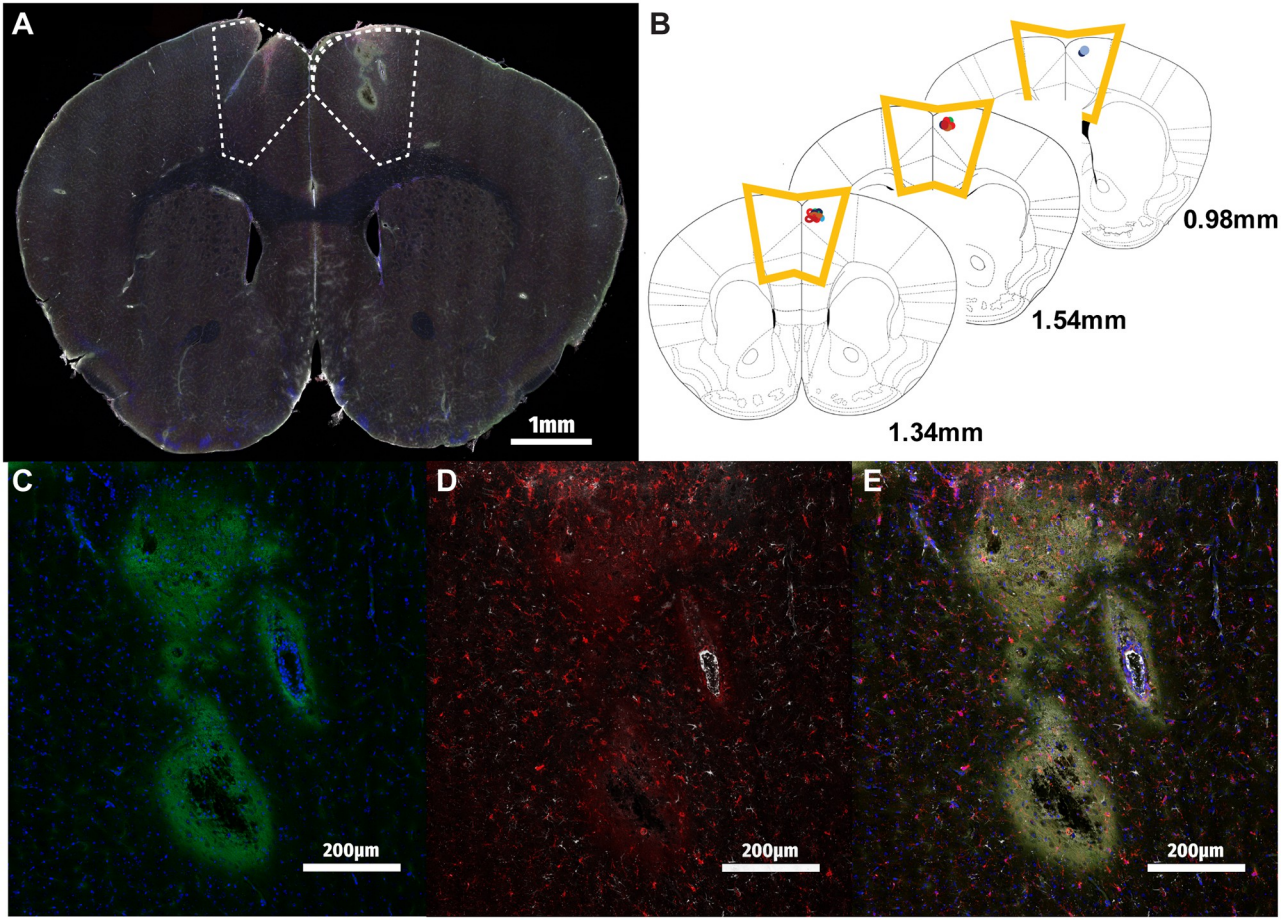
Detection of radioluminescent particles in mouse motor cortex. A) Tile-scanned merge of RLP-injected mouse brain at 40x zoom DAPI (blue), RLPs (green), IBA-1 (red) and GFAP (white). M2 region highlighted with dotted line. B) Overlay of RLP injection sites determined by sectioning M2 region of n = 12 animals demonstrates highly reproducible localization of RLP delivery. C) RLP injection site inset demonstrates fluorescein signal near the injection site. D) IBA-1 expressing microglia and GFAP-expressing astrocytes are detectable by the injection site 24 hours post-injection. E) RLP and cellular markers together.

To confirm that stereotaxically microinjected RLPs were present in measurable quantities at the target region, consecutive coronal sections spanning the anterior-posterior extent of the M2 region were prepared and visualized using confocal microscopy. Fig 3A demonstrates a representative image of a full coronal brain section, highlighting the site of the RLP injection in the right hemisphere. Cross-referencing the locations of RLP injection sites as determined by microscopy with a reference anatomic atlas demonstrated highly accurate and reproducible delivery of the RLP payload to M2 across all experimental animals (Fig 3B) [27].

Closer examination of the injection site under greater magnification shows us the localization of RLPs as well as immune cells following injection into M2. RLP spread was detected by visualization of fluorescein adjacent to the injection site, in the path of the needle superficial to the injection site, as well as surrounding nearby vasculature (Fig 3C). Brain sections were additionally stained for the astrocytic (GFAP) and microglial (IBA-1) cell markers to assess the involvement of these immune cells in the response to RLPs, as shown in the inset 24 hours post-injection (Fig 3D and 3E). Overall, we were able to determine here that RLPs can be injected reliably in secondary mouse motor cortex and fluorescently detected, alongside markers of astrocytes and microglia.

### Variation of RLP-associated fluorescence with dose and time

**Fig 4:**
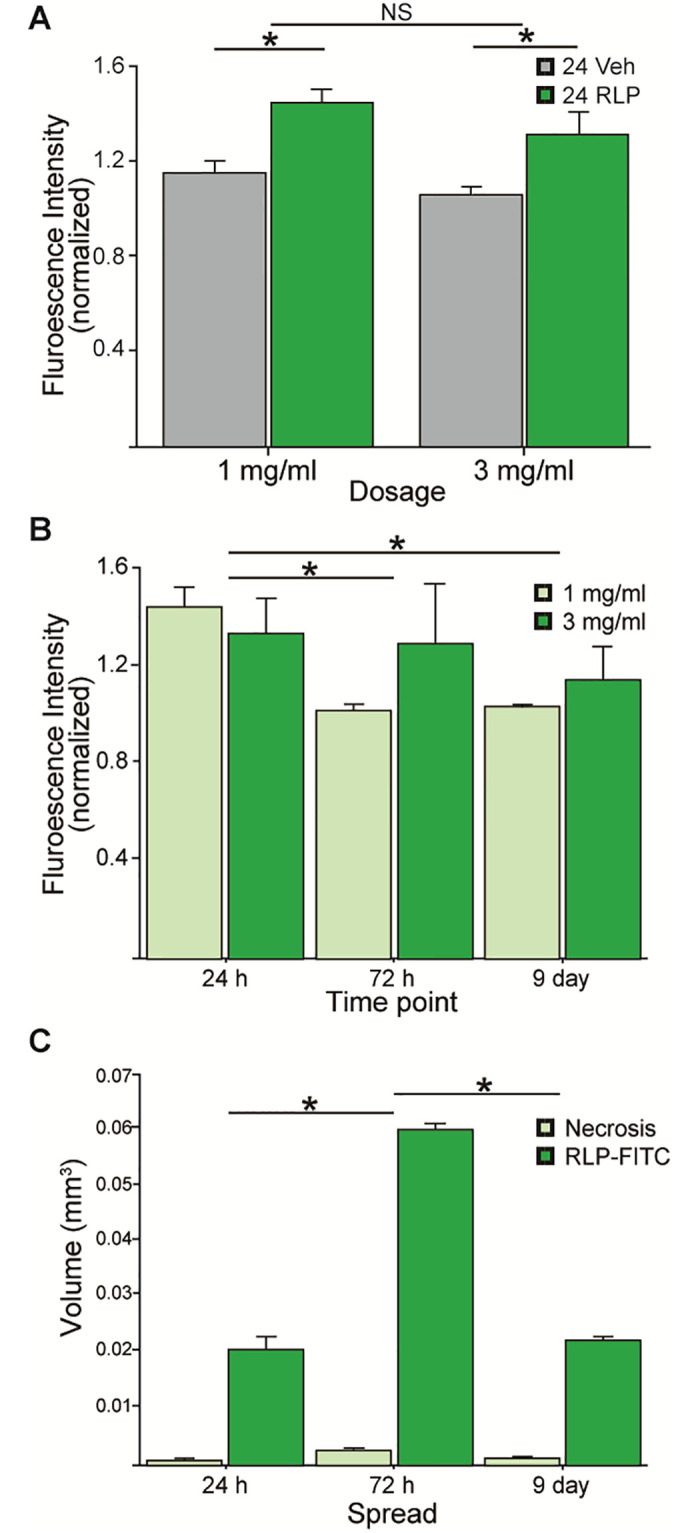
Quantifying changes in radioluminescent particle signal over time. A) mean background-normalized fluorescence intensity of the fluorescein (FITC) channel across the entire M2 region compared to the contralateral saline vehicle-injected side was significantly greater (p<0.05), whereas no differences were observed between 1mg/ml and 3mg/ml groups (p>0.05). B) Time course data showing control-normalized RLP-fluorescein (FITC) fluorescence intensity at 1mg/ml and 3mg/ml doses at each 24hr, 72hr and 9 days post-injection. Significantly greater fluorescence was observed in the 24hr group compared to the 72hr and 9 day timepoints (p>0.05). C) The volume of RLP spread and the area of necrosis for each 24hr, 72hr and 9 day time points in 1 and 3mg/ml treated animals.

After determining that RLPs can be reproducibly delivered stereotaxically and visualized in mouse motor cortex, aspects of RLP fluorescence intensity over time were investigated. Our initial experiments demonstrated that endogenous autofluorescent background in the fluorescein emission spectrum was notable across the imaged cortex as noted by others [28, 29], so we first established that signal in this spectrum was indeed greater overall in RLP-injected M2 regions as compared to their vehicle-injected contralateral regions (Fig 4A; p<0.05). Interestingly, there was no difference in the amount of RLP signal detected over background, with the 3 mg/ml injected brain sections when compared to the 1 mg/ml dose (Fig 4A, p>0.05 “NS”), suggesting a limit to the sensitivity of this particular fluorophore.

By sacrificing animals carrying RLPs at different intervals post-injection, we sought to determine the rate of RLP signal dissipation over time, as a proxy for measuring the clearance of RLPs from the site. We found a significant effect for time, with the 24 hour group showing significantly greater amount of fluorescence as compared to the 72 hour and 9 day groups, respectively (Fig 4B; p<0.05). Interestingly, no dose effect was found in either of the latter time periods (p>0.05); although it appears that the brain regions that received 3 mg/ml showed greater signal persistence over time, this effect was not determined statistically significant (p>0.05).

Lastly, by manually outlining the extent of RLP signal as well as region of dead tissue at the injection site, we quantified the volume of RLP spread over the time points investigated (Fig 4C). We found that 72 hours post-injection the RLP-associated fluorescent signal occupies approximately threefold the volume as compared to 24 hours and 9 day post-injection, indicating a significant initial diffusion over the first days (p<0.05), followed by partial clearance of the RLPs by the 9 day time point. Throughout the entirety of the experiment, the area of tissue death caused by the injection was measurable, but minimal as compared to the extent of RLP spread throughout the motor cortex. Taken together, these measurements establish that RLPs injected into the M2 region are readily detectable up to 9 days post-injection, that RLPs diffuse through the brain ECM for several days before partial clearance is observed, although overall RLP signal intensity falls significantly after 24 hours.

### Time evolution of immune cell responses to RLP injection

**Fig 5:**
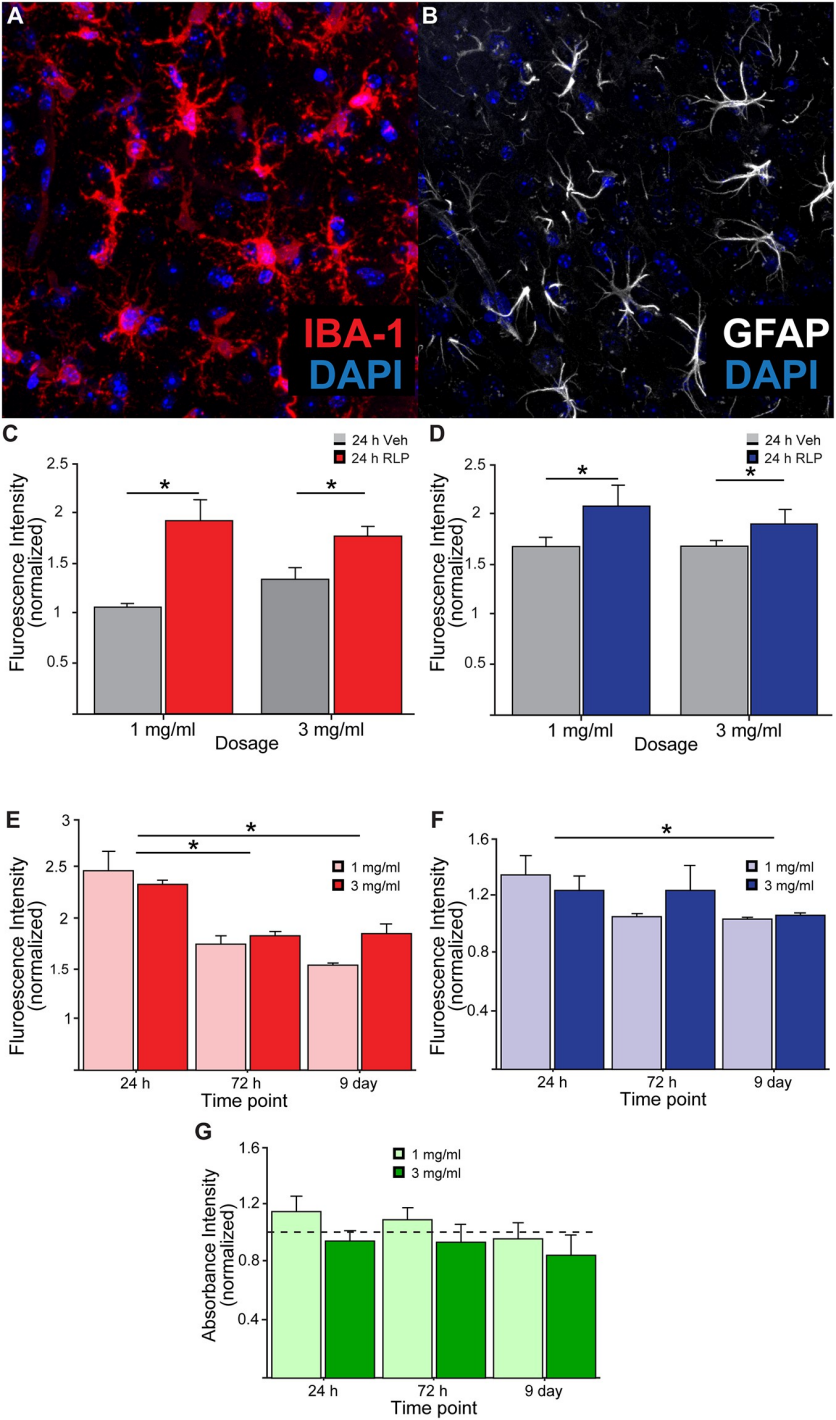
Quantifying immune cell presence adjacent to radioluminescent particle injections. A) Representative image of IBA-1^+^ microglia and B) GFAP^+^ astrocytes near RLP injection site. C) Fluorescence intensity of IBA-1 and D) GFAP staining in vehicle (saline) versus RLP-injected M2 following injections of 1mg/ml and 3mg/ml in mice sacrificed 24 hours after injection. E) Total fluorescence intensity of IBA-1 and F) GFAP in RLP-injected M2 (normalized to vehicle-treated contralateral hemisphere) for animals receiving 1 and 3 mg/ml concentrations of RLPs 24 hours, 72 hours, and 9 days post-injection. (G) *In vitro* Alamar blue assay revealed no significant changes in cell viability of cultured neurons following RLP exposures at multiple timepoints compared to untreated controls (dashed line at “1”).

To assess the anticipated recruitment of immune cells to the site of the RLP injection, brain sections were additionally immunostained for the astrocytic marker glial fibrillary acidic protein (GFAP) as well as the microglial marker allograft inflammatory marker 1 (IBA-1), and both were found to be significantly elevated in the RLP group over time (Fig 5A and 5B; p>0.05). Similar to the previous RLP analyses, total fluorescence intensity for each IBA-1 and GFAP was quantified across M2 at both 1 and 3 mg/ml RLP concentrations 24 hours, 72 hours, and 9 days post-injection. Recruitment of both GFAP^+^ and IBA-1^+^ cells to mouse motor cortex in the RLP injected hemispheres was detectable for both RLP concentrations, but interestingly no dose effect was found to be significant, indicating some tolerance to the higher concentration of RLPs tested (Fig 5C and 5D; p>0.05).

Despite overall similarities in the extent of recruitment of GFAP^+^ and IBA-1^+^ cells to the injection site 24 hours after RLP injection, immune cell presence over time reveals differential dynamics between the two cell populations. IBA-1 fluorescence intensity in M2 (normalized to vehicle treated contralateral M2 region) was significantly reduced between 24 and 72 hours post-injection (p>0.05, while GFAP signal was unchanged until 9 days after the injection (Fig 5E and 5F; p>0.05). Interestingly, no difference between either immune cell marker intensity was noted between the 1 and 3 mg/ml doses at any of the time points tested, although GFAP signal appears to trend back down to baseline more rapidly in animals receiving 1 mg/ml RLPs. At the end of the 9 day experimental period IBA-1 signal intensity remained above that of the vehicle treated contralateral hemisphere (Normalized Intensity>1), while the GFAP signal largely returned to basal levels, highlighting the need for more detailed study of the dynamics of immune cell response for RLPs to be considered as an injectable light delivery system.

To determine whether RLPs had direct toxic effects to neurons we exposed primary hippocampal neurons cultured from P0 mouse pups to RLPs at the same concentrations and time periods as were used in vivo (1mg/ml and 3mg/ml; 24, 72, and 216 hours, respectively). To synchronize the endpoint assay at 15 days in vitro (DIV) in culture, we staggered the initial particle treatments, beginning at DIV 14, DIV 12, and DIV 6, respectively. We then used alamarBlue assay, which utilizes the reducing environment of live cells as a readout of metabolically active cells to determine viability across treatment conditions. Fig 5G shows normalized absorbance values of four replicates across the three timepoints in conditions with particles relative to control cells with no particles (indicated by the dashed line at 1). We found no significant differences between any groups indicating that cells across all timepoints and particle conditions were able to metabolize resazurin equivalently. Thus, we found no evidence that treatment of neurons in vitro with RLPs caused direct impairments to cell viability.

## Discussion

From drug delivery to biodetection and optical stimulation, nanoparticles promise to serve as a platform technology for a new wave of preclinical and clinical research initiatives [30]. Inherent material properties of nanoparticles such as scintillation can be taken advantage of and paired with diverse potential modifications to surface chemistry, leading to broad potential utility of such particles for researchers [31, 32]. Material science groups synthesizing scintillating nanoparticles for biomedical applications frequently model dispersion of particles *in silico* or in polymers, gels, and fluids, but relatively few reports published to date include observations of these nanoparticles *in vivo* [33–35]. The life sciences community has begun more recently to describe the characteristics of the interactions between novel nanoparticles and biological systems, but variability in nanoparticle size, composition and surface chemistry still generate a wide heterogeneity of results [36–38]. In this study we report the synthesis of a novel BSA-coated, fluorescein-conjugated Y2(SiO_4_)O:Ce nanoparticle (RLP) that is detectable in post-mortem mouse secondary motor cortex up to 9 days following intra-cranial injection, which elicits a small but significant transient recruitment of immune cells. In agreement with previous reports, the sub-200nm size of the RLPs reported here readily diffused through the brain extracellular space over the first 72 hours post-injection, before partial clearance at 9 days [33]. Such a tool could eventually serve the basis for a biologically-tolerated injectable light delivery system for use in optogenetic manipulations to brain regions in a manner far less invasive than previous surgical interventions.

If direct injection of RLPs proves to be a method of light delivery for optogenetic experiments less invasive than intra-cranial LED implantation, the logical next question lies in the particles’ ability to mediate meaningful biological outputs. One of the few studies on the topic to date by French *et al*. observed that light emitted from Y2(SiO_4_)O:Ce nanoparticles in response to UV stimulation caused an increase in the frequency of spontaneous excitatory postsynaptic currents (sEPSCs) in CA1 pyramidal cells expressing the light sensitive opsin channelrhodopsin-2 (ChR2)—commonly used for optogenetic experiments [39–41]. Interestingly, the stimulation paradigm used for these experiments was insufficient to change sEPSC amplitude or evoke light-gated action potentials, consistent with the relatively small photocurrents induced. Small changes in synaptic function, including a small reduction in the frequency of sEPSCs and an increase in paired pulse ratio of evoked excitatory transmission indicates an effect of the nanoparticles on presynaptic release probability [42, 43], one likely mediator of the minor effects on synaptic function during RLP UV stimulation.

Interesting among our observations concerning the neuroimmune response to local injection of nanoparticle was the differing temporal regulation of GFAP and IBA-1-positive cell populations. The response of astrocytes to a stab wound in the cerebral cortex of rodents has long been appreciated [44–46]. While our results do not preclude an effect prior to 24 hours, and indeed, astroglial response to focal mechanical trauma can be detected within an hour of injury according to some reports, we did observe that the increase in GFAP staining had subsided by the 9 day time point [47]. Acute stab injury alone has been shown to induce astroglial presence only minimally in grey matter, however the inclusion of foreign material such a nitrocellulose, as reported by Balasingam *et al*., can induce robust recruitment and proliferation of astrocytes [48]. Once localized to the lesion, astrocytes are known to remodel the injury site itself in a number of ways, including alterations to blood-brain barrier permeability, glial scar formation as well as a number of changes to the tissue and extracellular matrix (ECM) structure and composition [49, 50]. These ECM alterations, primarily thought to facilitate clearance of cellular debris could underlie the diffusion of RLPs away from the injection site at the latter time points.
